# Editorial: Lived Culture and Psychology: Sharedness and Normativity as Discursive, Embodied and Affective Engagements with the World in Social Interaction

**DOI:** 10.3389/fpsyg.2020.00437

**Published:** 2020-03-11

**Authors:** Carolin Demuth, Pirkko Raudaskoski, Sanna Raudaskoski

**Affiliations:** ^1^Department Communication & Psychology, Centre for Qualitative Studies, Aalborg University, Aalborg, Denmark; ^2^Department Communication & Psychology, Mattering: Centre for Discourse & Practice, Aalborg University, Aalborg, Denmark; ^3^Faculty of Social Sciences, Centre for Social Research, Tampere University, Tampere, Finland

**Keywords:** culture, sharedness, normativity, discourse, embodiment, affect, social interaction

Understanding the cultural nature of human psychological functioning requires exploring the psychological means that bring about cultural forms of human conduct and experience. Cultural forms of perceiving and acting in the world are usually understood as being primarily rooted in socially *shared normativity*. However, it is rarely clear what exactly is to be understood as “sharedness” and “normativity” and what psychological means enable shared normativity. The Research Topic aims to contribute to a better understanding of these concepts by taking a closer look at discursive, embodied and affective engagements with the world.

Cultural psychologists agree that humans develop as *participants in cultural communities* (Rogoff, 2003) and that the way we perceive and understand the world is mediated through social interaction, primarily through semiotic sign systems such as language (Vygotsky, 1978; Wertsch, 1991; Valsiner, 2014). Social constructionists argue that is through discursive practices that we construct specific versions of social reality (Gergen, 1985; Harré, 2012). Language here is understood as an activity, as social practice including embodied and affective dimensions that go beyond mere verbal talk (Shotter, 2008; Bertau, 2014). Language practices (“languaging”) and consciousness constitute each other (Vygotsky, 1978; Harré and Gillet, 1994; Linell, 2009) and constitute forms of life (Wittgenstein, 1953). Slunecko and Hengl (2007) describe this as language “‘owning' or ‘having' us,” arguing that humans are not simply beings who are disposed to language; rather, they are beings, who are acquired, modified, or formatted *by* language, and thus by their culture. (Geertz's, 1973) describes of “humans as animals suspended in webs of significance they themselves have spun” and culture as the symbolic “fabric of meaning in terms of which human beings interpret their experience and guide their actions” (p. 145). Developing this idea further, Brockmeier (2012) argues that it is through language—particularly narrative—that we are weaving this symbolic fabric (p. 442).

Looking merely at discursive practices in terms of verbal talk, however, sidelines the relational-affective nature of languaging, as well as other embodied aspects of social interaction. As Goodwin (2000, 2013) has convincingly shown, discursive practices need to be understood as part of a complex, collective and cultural human activity composed also of bodies, material artifacts, and the space.

The contributions of this Research Topic aim to further develop these ideas and to shed light on the processes involved both in the sharedness of certain ways of understanding the world and the normative dimension of social life. These processes are conceived of as action based, mutually shaped, dynamic and fluid, ever evolving, and situated in ecologically embedded social interaction. With this Research Topic we also intend to go beyond mere theoretical discussions and to illustrate how shared normativity can be empirically studied.

Larrain and Haye develop a theoretical argument about human psychological life as part of a living process of becoming by laying out a discursive and aesthetic view that takes the phenomenological experience of self into account. Karsten and Bertau develop a theoretical argument on how ideas come into being and convincingly lay out how thinking is social, embodied, and dialogically organized because it is entangled with language.

Trying to understand cultural aspects of experience and human conduct inevitably invites taking a developmental perspective to studying how shared normativity is enacted in interactions with children. Several contributions stress the role of affect in these processes. Forrester pinpoints the shortcomings of common discursive approaches to address human affect and emotion. He proposes that psychoanalytical thinking might inform our understanding of how socially shared normativity emerges during infancy and early childhood. Fantasia et al. address shared normativity by studying the relational dynamics in interactions of mothers suffering from postpartum depression with their infants. Their findings challenge traditional views on “intrusiveness” as based on specific individual behaviors and suggest that what hinders mutual coordination in these interactions is the absence or violation of interactional norms.

Cekaite and Ekström and Cekaite and Andrén studied emotion socialization practices in Swedish preschools using micro-analytic multimodal video analysis. They identified specific communicative practices through which the expression of negative emotions is responded to as well as how laughter functions as an intricate process of inviting others into the common emotional and experiential ground. The studies shed light on the varied societal circumstances for learning and developing the norms and values that are communicated through these practices. In a similar vein, Takada studied the use of the term hazukashii (indicating shamefulness or embarrassment) in caregiver interactions with small children in Japanese families. His findings reveal that the term was commonly used to frame an action or act as inappropriate in a given context, but also to frame an activity as teasing and promoting a cooperative and pleasant atmosphere. Wiggins' paper discusses how the enjoyment of food and the sharing of mealtimes become a normative cultural and social practice by studying video-taped infant mealtimes in families in Scotland within a discursive psychology framework. Her findings reveal that eating enjoyment can be considered as much an interactional achievement as an individual sensation. Sirota's study looks at how children in U.S. middle class families in California are apprenticed into perceiving, appraising, and reacting to the emotions of self and others as cultural indicators for proper comportment.

From a slightly different perspective, Aarsand investigated digital literacy practices in children's everyday lives at a Norwegian preschool. His findings shed light on how digital media become part of how children are instructed to experience, interpret, understand and act in the world.

Raudaskoski and Klemmensen discuss the “turn to affect” as assemblage and emergence, and propose how linkages between episodes of affect as embodied social practice can be traced by drawing on Goodwin's multimodal ethnomethodological conversation analysis (EMCA) when studying institutional interactions with people who have an acquired brain injury.

All together, these papers provide a deep discussion of shared normativity as rooted in social interaction by considering its discursive, embodied, affective nature embedded in a material world. They also provide concrete suggestions for how to analyze these concepts empirically.

## Author Contributions

All authors listed have made a substantial, direct and intellectual contribution to the work, and approved it for publication.

### Conflict of Interest

The authors declare that the research was conducted in the absence of any commercial or financial relationships that could be construed as a potential conflict of interest.
